# Supplementary material for: Vectorial lasing with designable topological charges based on Möbius-like correspondence in quasi-BICs
Source: Light Sci Appl. 2026 Mar 30;15:184. doi: 10.1038/s41377-026-02269-7 (PMC13033454; doi:10.1038/s41377-026-02269-7)
Supplement: Supplementary file 1 — Supplementary Information [file 41377_2026_2269_MOESM1_ESM.pdf]

Supplementary Information for Vectorial lasing with designable topological charges based on Möbius-like correspondence in quasi-BICs

Xinhao Wang[1,†], Zhaochen Wu[1,†], Jiajun Wang[1,2,3,†,*], Lei Shi[1,2,3,4,5,*], and Jian Zi[1,2,3,4,5,*]

[1]State Key Laboratory of Surface Physics, Key Laboratory of Micro- and Nano-Photonic Structures (Ministry of Education) and Department of Physics, Fudan University, Shanghai 200433, China.
[2]Shanghai Research Center for Quantum Sciences, Shanghai 201315, China.
[3]Shanghai Key Laboratory of Metasurfaces for Light Manipulation, Fudan University, Shanghai 200433, China.
[4]Institute for Nanoelectronic Devices and Quantum Computing, Fudan University, Shanghai 200438, China.
[5]Collaborative Innovation Center of Advanced Microstructures, Nanjing University, Nanjing 210093, China.
[†]These authors contributed equally: Xinhao Wang, Zhaochen Wu, Jiajun Wang.
[*]e-mail: jiajunwang@fudan.edu.cn; lshi@fudan.edu.cn; jzi@fudan.edu.cn

Table S1: Comparison with other representative vectorial lasing generation

Reference		Type	Prinpciple	Passive/Active	Topological charge	Size
1	Appl. Phys. Lett. 20, 266–267 (1972)	Intracavity conversion	Birefringent crystal	Active	+1	Bulky system
2	Applied Optics 29, 2234–2239 (1990)	Intracavity conversion	Interference of linearly polarized modes	Active	-1, +1	Bulky system
3	J. Phys. D: Appl. Phys. 32, 2871 (1999)	Intracavity conversion	Polarization selective mirrors	Active	+1	Bulky system
4	Appl. Phys. Lett. 77, 3322–3324 (2000)	Intracavity conversion	Coherent summation of two orthogonally polarized modes	Active	+1	Bulky system
5	Opt. Communications 203, 1–5 (2002)	Few mode fiber laser	Mode selection in multi-mode fiber	Passive	-1, +1	Bulky system
6	Opt. Letters 27, 285-287 (2002)	Free space conversion	Space-variant subwavelength grating	Passive	-2, -1, +1, +2	Bulky system
7	Opt. Letters 30, 3063-3065 (2005)	Intracavity conversion	Conical Brewster prism	Passive	+1	Bulky system
8	Opt. Express 14, 2650-2656 (2006)	Free space conversion	Spatial light modulators	Passive	+1	Bulky system
9	Opt. Communications 281, 732–738 (2008)	Free space conversion	Segmented spatially variant λ/2 plates	Passive	+1	Bulky system
10	Nano Lett. 13, 4269–4274 (2013)	Free space conversion	Detour-phase hologram	Passive	+1	~40 × 40 μm^2
11	ACS Photonics 3, 1558-1563 (2016)	Free space conversion	Metasurfaces	Passive	+1	Micron scale
12	Nat. Photonics 14, 498-503 (2020)	Intracavity conversion	Metasurfaces	Active	+1	Bulky system
13	Nat. Photonics 16, 359-365 (2022)	Intracavity conversion	Metasurfaces	Active	+1	Bulky system
14	Nat. Commun. 13, 7795 (2022)	Conversion on VCSEL	Metasurfaces	Active	+1	~100 × 100 μm^2 Height ~630 μm
15	Adv. Mater. 35, 2204286 (2023)	Conversion on VCSEL	Metasurfaces	Active	+1, +2, +3	~120 × 120 μm^2
16	Nat. Photonics 13, 283-288 (2019)	DBR cavity	Benzene-like photonic molecules	Active	-2, +1	~7 × 7 μm^2 Height 8 μm
17	Nat. Nanotechnol. 15, 1012-1018 (2020)	Topological cavity	Dirac-vortex topological cavity	Active	+1, +2, +3, +4	~50 × 50 μm^2
18	Nature Photonics 16, 279-283 (2022)	Topological cavity	Dirac-vortex topological cavity	Active	+1	~500 × 500 μm^2
19	Phys. Rev. Lett. 125, 013903 (2020)	Topological cavity	Spin-Momentum-locked edge mode	Active	+2	~13 × 8 μm^2
20	Nature Photonics 18, 286-293 (2024)	Optical cavity	Photonic disclination cavity	Active	-1, +1	~3 × 3 μm^2
21	Nature 612, 246-251 (2022)	Micro-ring cavity	Whispering gallery modes, Non-Hermitian photonics	Active	-2 to +2	~150 × 300 μm^2
22	Science 367, 1018-1021 (2020)	Photonic crystal	BIC	Active	+1	~40 × 40 μm^2
23	Nat. Commun. 13, 6485 (2022)	Photonic crystal	BIC	Active	-1	~74 × 74 μm^2
24	Phys. Rev. Lett. 134, 133802 (2025)	Photonic crystal	BIC	Active	-2, +1	~150 × 150 μm^2
25	Nat. Commun. 15, 9544 (2024)	Plasmonic quasicrystal	Symmetry design based on gruop theory	Active	-3, -4, -5, +7, -17, -19	Diameter 290 μm
This work		Compound cavity	Quasi-BIC, Möbius-like correspondence	Active	-5 to +5	~150 × 150 μm^2

Section 1. Details on the finite-size simulation

The far-field profile originates from the radiative components of the near field and reflects the radiation characteristics of the cavity mode. These two profiles can be related through a Fourier transformation. Here, we would like to provide more information and discussion for the simulated near-field and far-field profiles of the cavity modes presented in Fig. 3 of the main text.

As described in the Materials and methods section, the near-field profiles ($|E|^2$) were calculated using the finite-element method by modeling a finite-size PhC structure, as illustrated in the middle panels of Figures S1**a** and S1**b**. Taking the central plane of the Si_3N_4 layer as the $z = 0$ reference, we extracted the field distribution on an $x - y$ plane at $z_1 = 200$ nm to represent the near-field profile (left panels of Figures S1**a** and S1**b**, consistent with Fig. 3 in the main text). For comparison, another $x - y$ plane at $z_2 = 900$ nm was analyzed and presented in the right panels of Figures S1**a** and S1**b**. After propagating along the z-direction for this distance (approximately 1.5 wavelengths), the field profile undergoes a significant evolution. This is because the non-radiative components near the slab (at $z_1 = 200$ nm) cannot propagate along the out-of-plane direction.

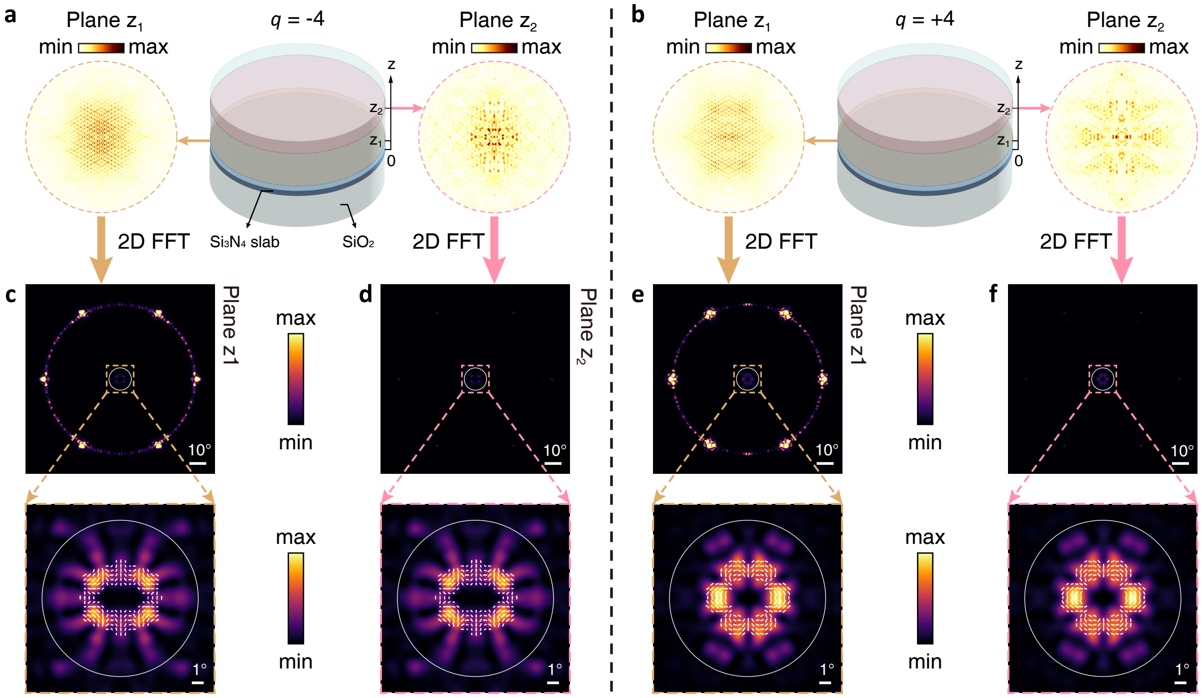

Figure S1: **a**–**b**, Schematic illustrations of the finite-size PhC cavity models for vectorial lasing with topological charges of −4 (**a**) and +4 (**b**). Two x–y planes are selected to display the real-space field distributions. Each panel is plotted with an independent intensity scale. **c**–**f**, Fourier-transformed (FT) spectra of the real-space field distributions in **a** and **b**. The white circular lines denote the light cone, and the dashed squares highlight the central regions of the FT spectra, whose detailed field and polarization distributions are shown in the lower panels.

The radiation behavior can be further visualized by applying a two-dimensional Fourier transformation to the real-space field distribution in $x-y$ plane [1, 2, 3]. Figures S1**c-f** present the Fourier-transformed (FT) spectra of the real-space distributions in **a** and **b**. The white circular line marks the light cone: plane wave components of **k**-vectors inside the circle correspond to radiative components that can couple to the far field, whereas those outside represent non-radiative components. For the FT spectra of z_1 plane (left panels in Figures S1**a** and S1**b**), a large portion of the field lies outside the light cone, explaining the difference between near- and far-field profiles. For z_2 plane (right panels in Figures S1**a** and S1**b**), which is located further above the Si_3N_4 slab, these non-radiative components vanish, as evidenced by the significant reduction of the plane wave components outside the light cone in the corresponding FT spectra. For direct comparison, the panels in Figures S1**c-d** (S1**e-f**) share the same intensity scale (maximum value). The radiative components inside the light cone are clearly presented in the lower panels of Figures S1**c-f**, where the calculated FT spectra and polarization distributions at both planes exhibit nearly identical far-field features. In the simulations shown

in Fig. 3 of the main text, we used the real-space fields at z_2 to perform the Fourier transformation, ensuring that only radiative components contribute to the obtained far-field profiles and polarization characteristics.

Section 2. Details on the sample fabrication

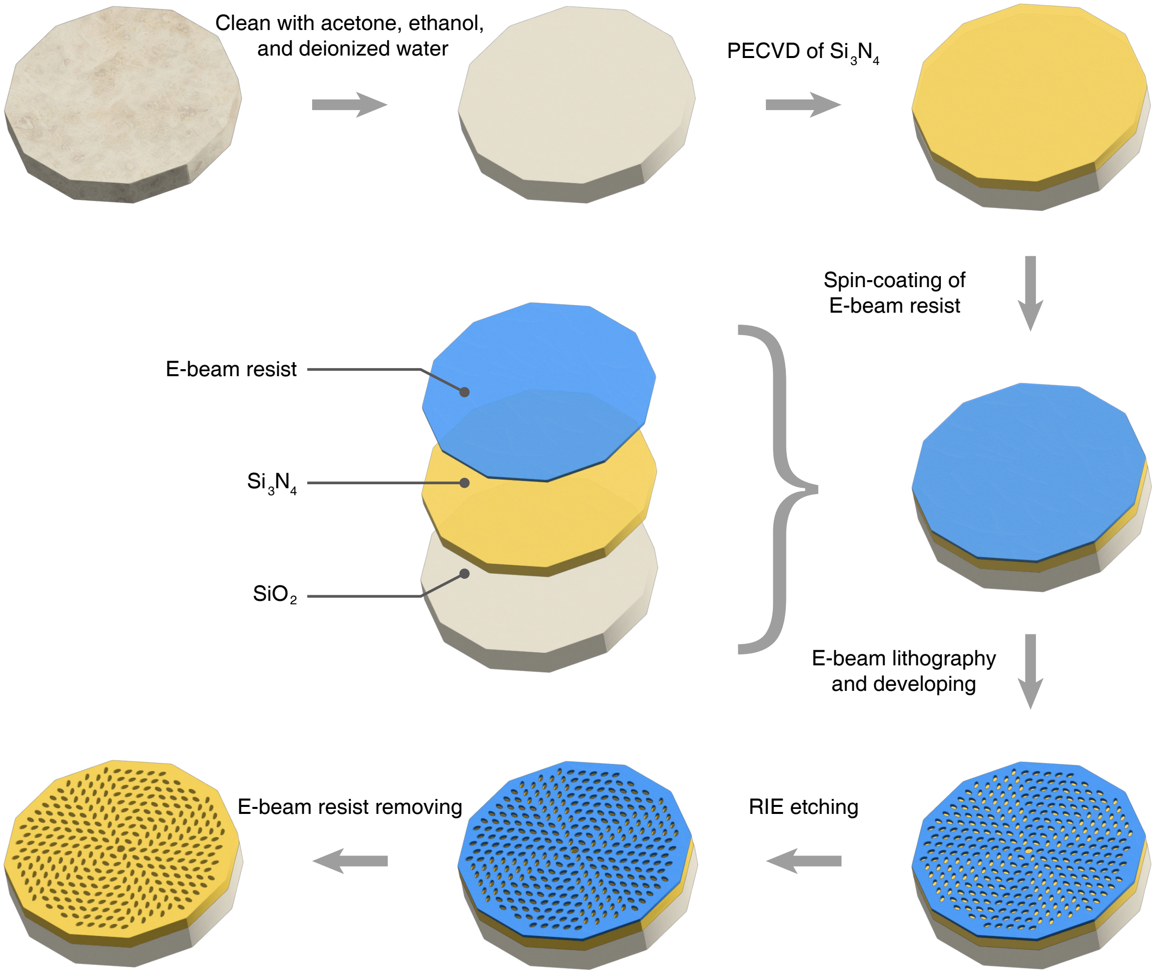

Figure S2: Fabrication process of the sample for vectorial lasing.

Figure S2 illustrates the fabrication process of the PhC structure for vectorial lasing, as described in the Materials and methods section. The morphology of the PhC structure is primarily defined through electron-beam lithography (EBL) and reactive ion etching (RIE). In the fabrication process, the EBL step ensures accurate control of the major-axis orientation (θ) of the elliptical holes, while slight deviations may occur in the exact values of the major axis (b_1) and minor axis (b_2). During the subsequent RIE process, the etching primarily affects the axial lengths of the ellipses but does not alter their orientation. The variations in the PhC structural parameters are summarized in Figure S3**b**. For more precise control of the elliptical-hole dimensions, multiple fabrication iterations can be performed experimentally to calibrate the actual changes of Δb_1 and Δb_2 during processing and adjust the setting parameters b_{10} and b_{20} accordingly, ensuring that the final structure closely matches the target geometry.

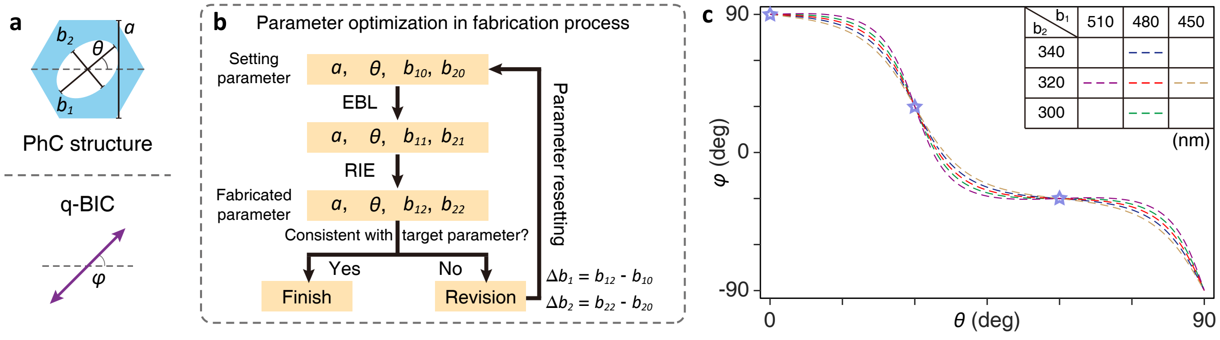

Figure S3: **a**, Definition of the PhC structural parameters and the polarization orientation of the quasi-BIC. **b**, Schematic illustration of parameter optimization during the fabrication process. The lattice constant (a) and orientation angle (θ) are precisely defined by EBL and remain unchanged during the RIE process, while the major (b_1) and minor (b_2) axes may experience dimensional variations. **c**, Influence of the elliptical-hole size variations on the Möbius-like correspondence of the quasi-BIC.

In practice, once iterative optimization yields structural parameters close to the design values, the residual deviations exert only a minor influence on the eigen-polarization of the quasi-BIC. Here, we therefore examine the impact of small dimensional errors on the quasi-BIC characteristics. To quantitatively evaluate this effect, we conducted numerical simulations by introducing controlled perturbations to the major and minor axes of the elliptical holes. The corresponding eigen-polarization states and Möbius-like correspondence were calculated and compared, as shown in Figure S3**c**. When a 6.25% dimensional perturbation was applied, the Möbius-like correspondence exhibited only slight deviations and remained unchanged at the nodes of $\theta = 0°$, $60°$, and $120°$, confirming the robustness of the designed quasi-BIC against small fabrication-induced variations.

Section 3. Details on optical measurement system

Figure S4 presents the schematic of the momentum-space spectroscopy measurement system employed for optical measurements. Based on the principle of Fourier optics, the optical lenses are used in the systems to perform optical Fourier transform to analyze the momentum-space [4].

The momentum-space imaging spectroscopy system operates in two modes: spectrometer mode and imaging mode. In spectrometer mode, a spectrometer is positioned with its entrance slit aligned to the Fourier plane, corresponding to the sample plane. This alignment allows for momentum resolution along the axis of the slit. As light passes through the entrance slit, it is dispersed by a diffraction grating, resolving the wavelength along the axis perpendicular to the slit. The light is then detected by a charge-coupled device (CCD) camera, enabling the simultaneous capture of momentum-resolved and wavelength-resolved spectra in a single shot. In imaging mode, a CCD camera is directly positioned at the Fourier plane relative to the sample, capturing the complete momentum-space beam profiles. With the integration of a polarizer, the system can further perform polarization-resolved measurements.

The system's switchable incident light sources facilitate measurements for both PL spectra and transmittance spectra. For the transmittance spectra measurements, a broadband white light source illuminates the samples embedded with DMSO solution, using the spectrometer mode. PL characterizations are conducted by exciting samples embedded with the gain medium IR-140-DMSO (5.3 mM concentration) at ambient temperature with a femtosecond pulsed laser system. The system employs a continuous-wave 532 nm green laser (Verdi V-10, Coherent) to seed a Mira 900 mode-locked Ti laser (Coherent), generating ultrashort pulses at 800 nm with a repetition rate of 76 MHz. These pulses are amplified by a Legend Elite amplifier (Coherent), producing an 800 nm femtosecond laser with a 1 kHz repetition rate and a pulse width of ∼100 fs. A long-pass filter (850 nm) is used to filter out the residual pumping laser. The PL spectra and polarization orientations of the q-BIC lasing are measured using the spectrometer mode, while momentum-space lasing images are captured in imaging mode with a linear polarizer for polarization resolution.

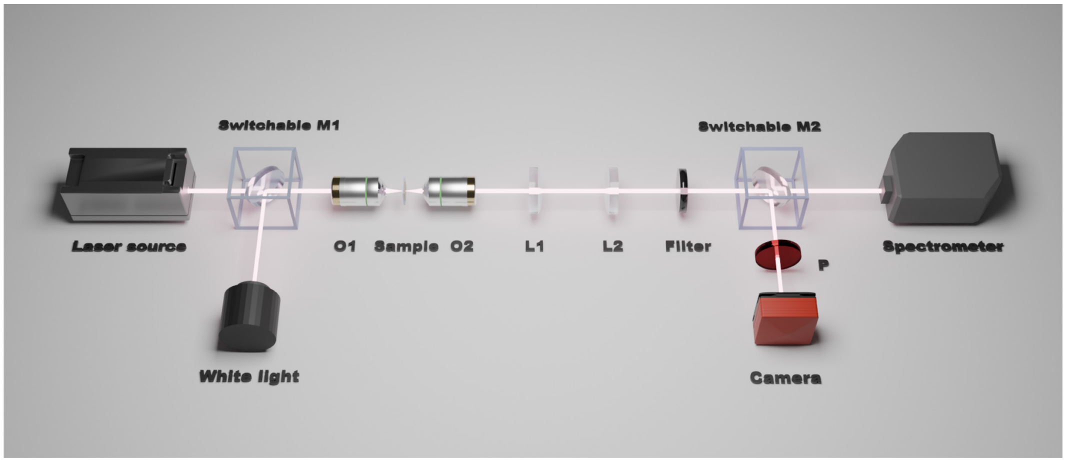

Figure S4: Schematic of the momentum-space imaging spectroscopy setup. O1, O2: objective lens; L1, L2: lens; M1, M2: mirror; P: linear polarizer.

Section 4. Experimental demonstration of the q-BIC with linear polarization

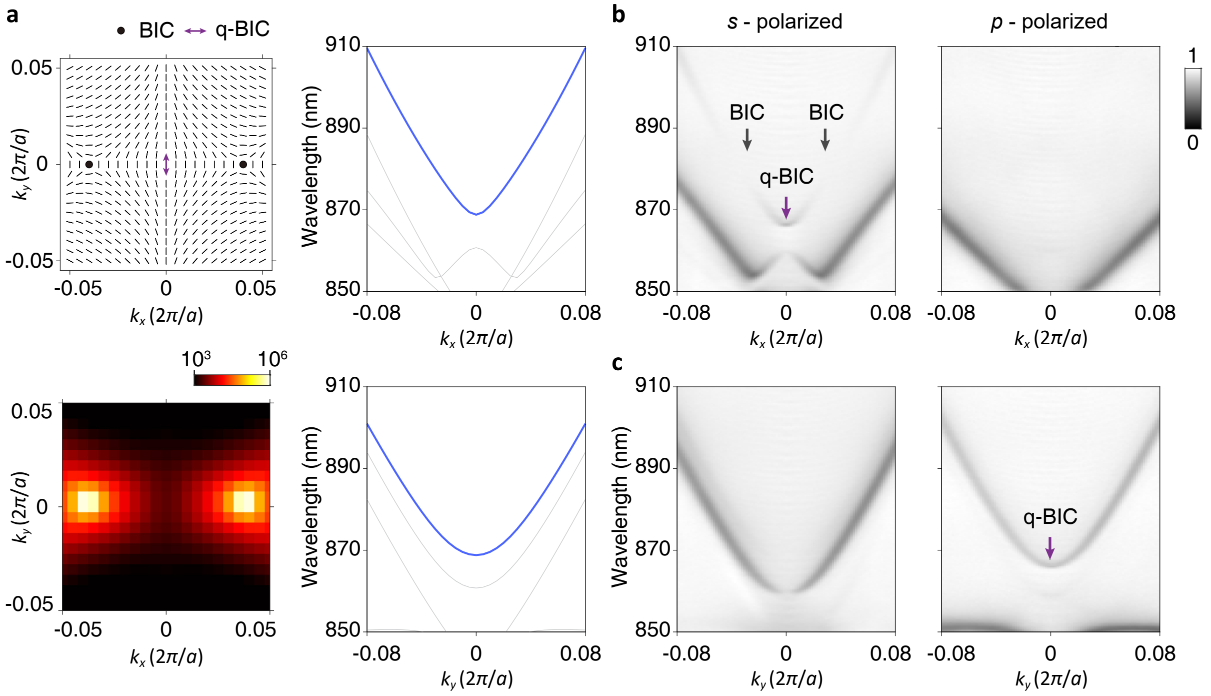

Figure S5: Linearly-polarized q-BIC of the C_2-symmetry PhC slab with $\theta = 0°$. **a**, Right, simulated photonic bands along the k_x direction (top) and k_y direction (bottom). The focused band with q-BIC used for lasing is highlighted in blue. Left, simulated momentum-space polarization field on the blue-marked photonic band and corresponding Q factor distribution. **b-c**, Experimentally measured in-plane momentum-resolved transmittance spectra of the fabricated C_2-symmetry PhC slab along the k_x direction (**b**) and k_y direction (**c**). The polarization-resolved spectra in the left (right) panels are measured under s-polarization (p-polarization) analysis. From the transmittance spectra, two off-Γ BICs are shown as the vanished regions on the photonic bands along the k_x direction, indicated by black arrows. The linearly-polarized q-BIC is observed at the Γ point, indicated by purple arrows.

Section 5. Details on the evolutions of polarization distributions

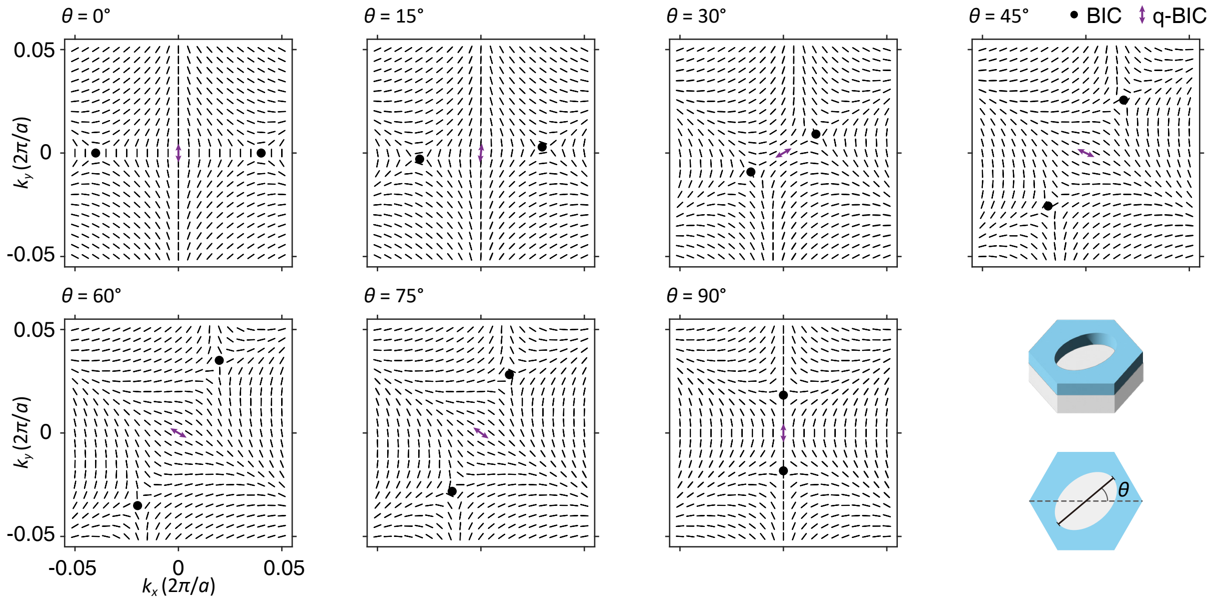

Figure S6: Simulated tunable evolutions of the polarization distributions under structural parameter manipulating. From the simulated polarization distributions of the C_2-symmetry PhC slabs with different θ, we can see that two off-Γ BICs and an at-Γ q-BIC are split from the BIC of -2 topological charge. The schematic of the structure is plotted on the lower right panel.

For these C_2-symmetry PhC structures, the rotation angle of the elliptical holes would cause slight changes in the frequencies of the q-BICs, as shown in Figure S7. The maximum wavelength deviation is about only 0.4 nm. Moreover, for the three PhC slabs used to construct the compound cavities in the main text (as shown in Figure S7**a**), their corresponding q-BICs exhibit identical frequencies.

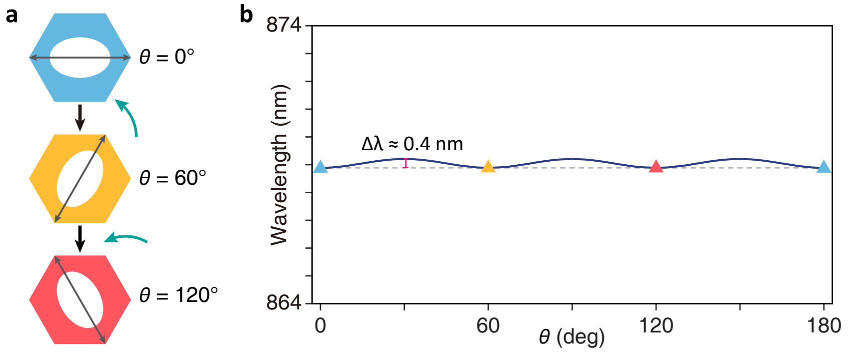

Figure S7: **a**, Schematic of the unit cells supporting q-BICs used for cavity construction in Fig. 3 of the main text. The unit cells can be transformed into one another by a 60° rotation. **b**, Wavelength variation of the q-BIC as a function of the rotation angle (θ) of the elliptical hole.

Section 6. Details on q-BIC lasing with different linear polarization

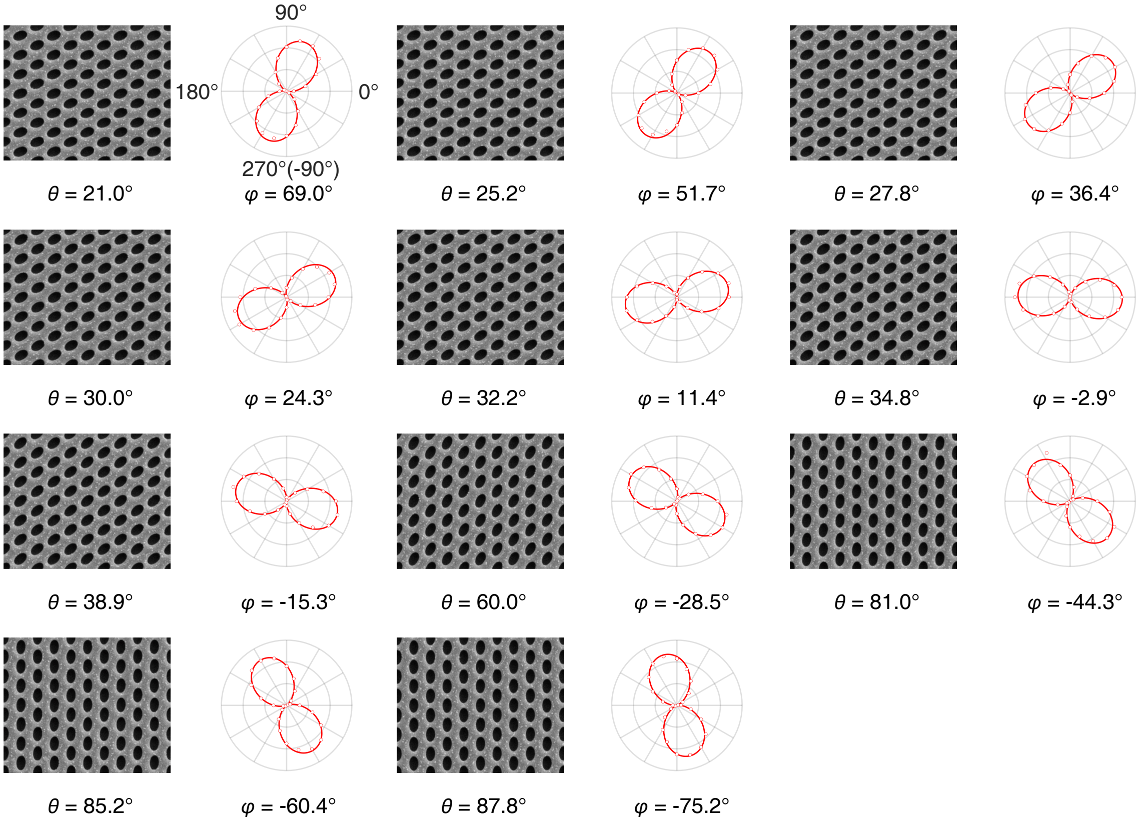

Figure S8: Experimentally measured relationship between lasing emission polarization and PhC structures' orientation. The SEM images and corresponding experimentally measured lasing emission polarizations are depicted in left and right of each panels, respectively.

Section 7. Details on the compound cavity design for vectorial lasing

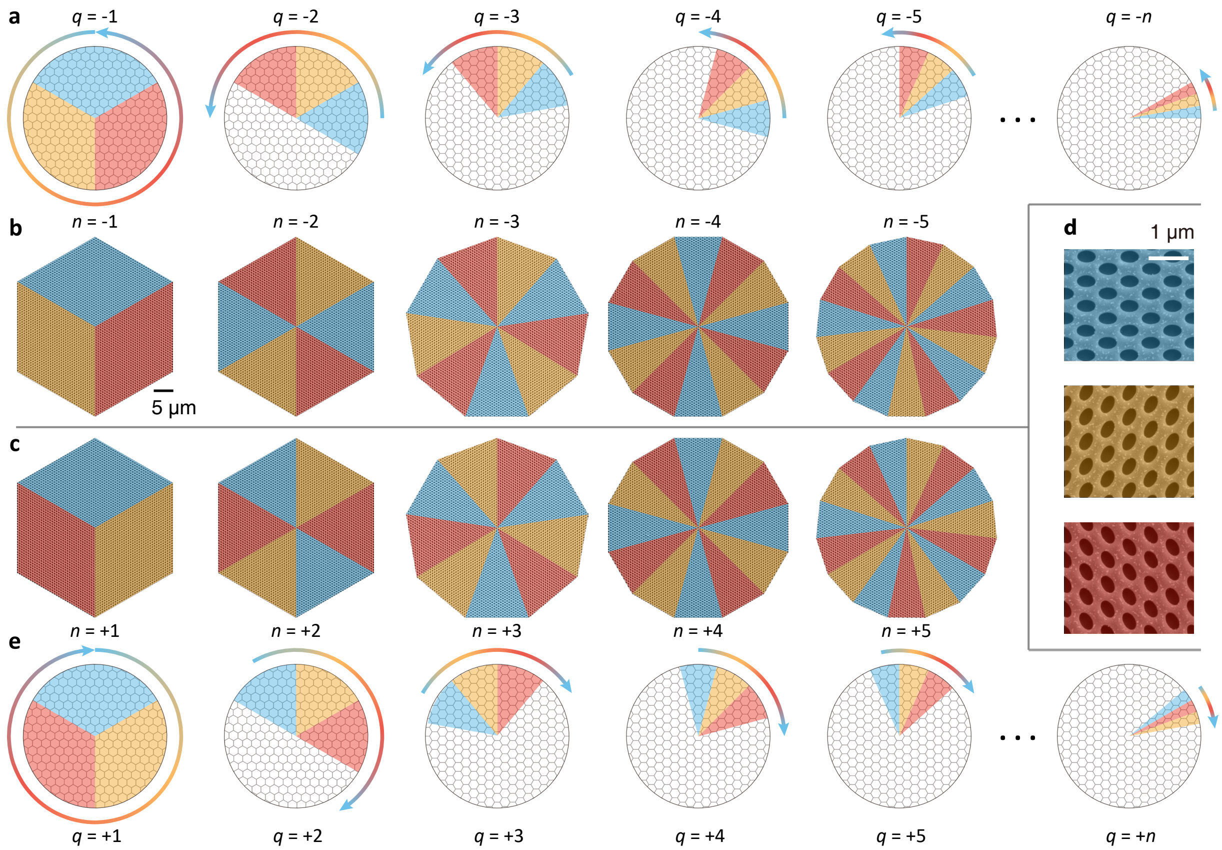

Figure S9: Design of compound cavities for vectorial lasing with topological charge ranging from −5 to +5. **a**, Schematics of the compound cavity design with negative topologcial charge. **b-c**, SEM images of the fabricated PhC structures for different charges. **d**, Detailed SEM images of quasi-BIC PhC slabs. Different colors are plotted to highlight the difference in structural parameter (blue: $\theta = 0$, yellow: $\theta = \pi/3$, orange: $\theta = 2\pi/3$). **e**, Schematics of the compound cavity design with positive topologcial charge.

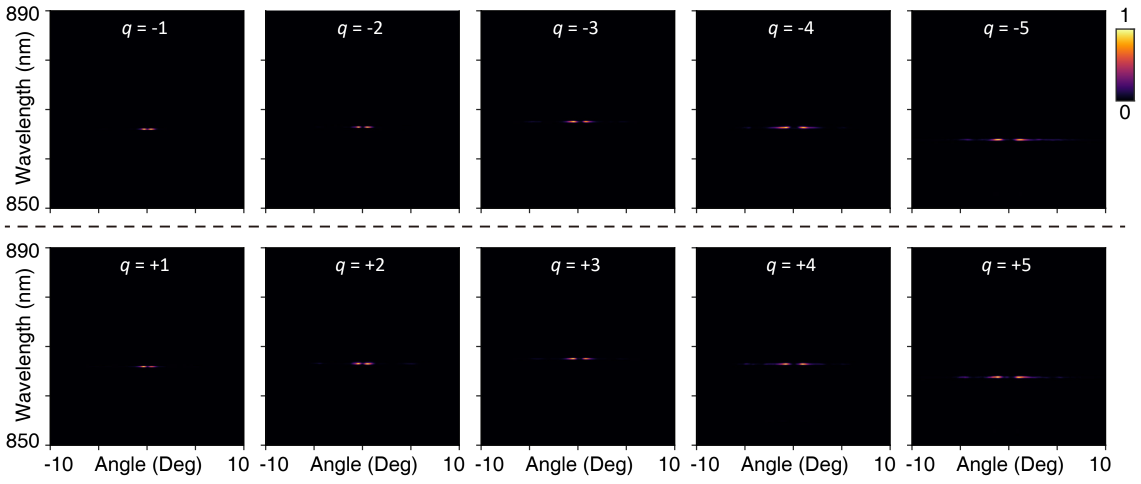

Figure S10: Measured angle-resolved lasing spectra (above threshold) of the fabricated compound cavities supporting vectorial lasing with different topological charges in the main text.

Section 8. Measured polarization-resolved lasing images

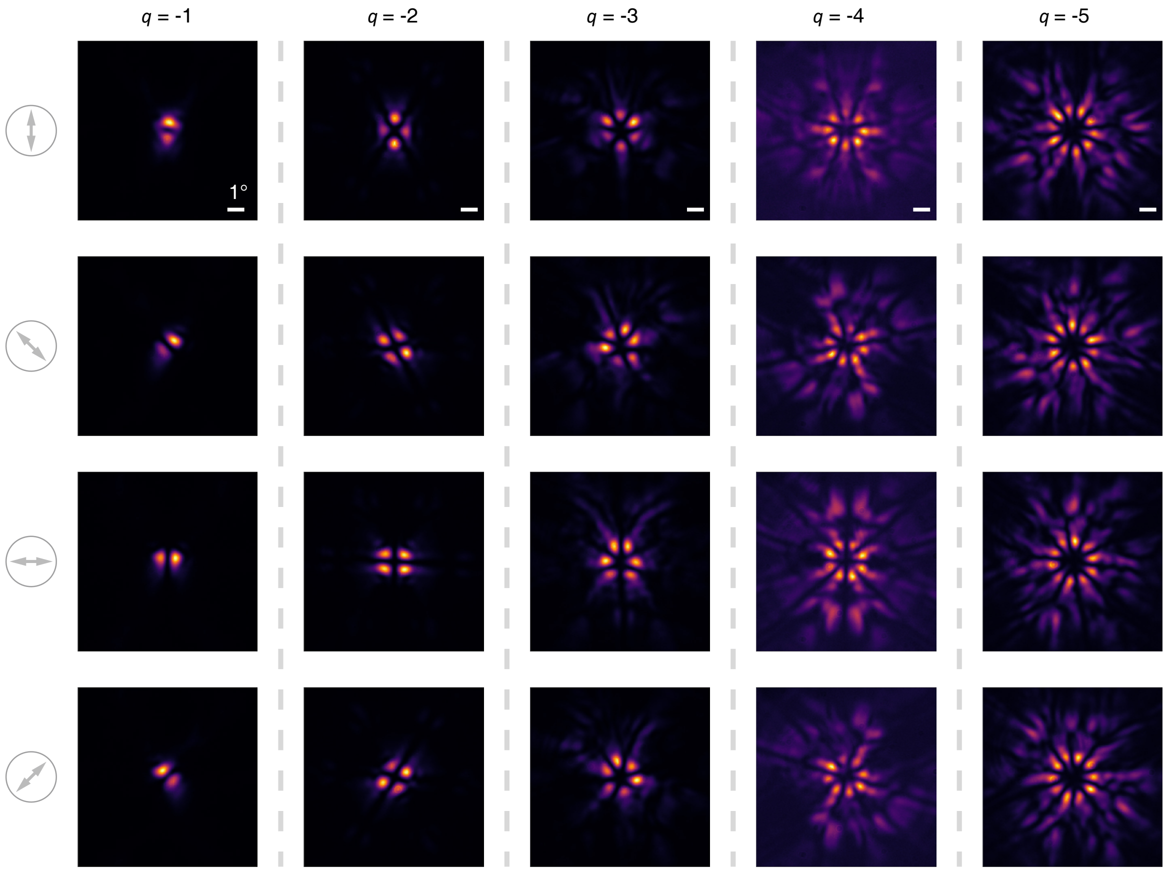

Figure S11: Measured polarization-resolved lasing images in momentum space with topological charges ranging from -1 to -5. The left grey arrows show directions of the analyzed polarizations.

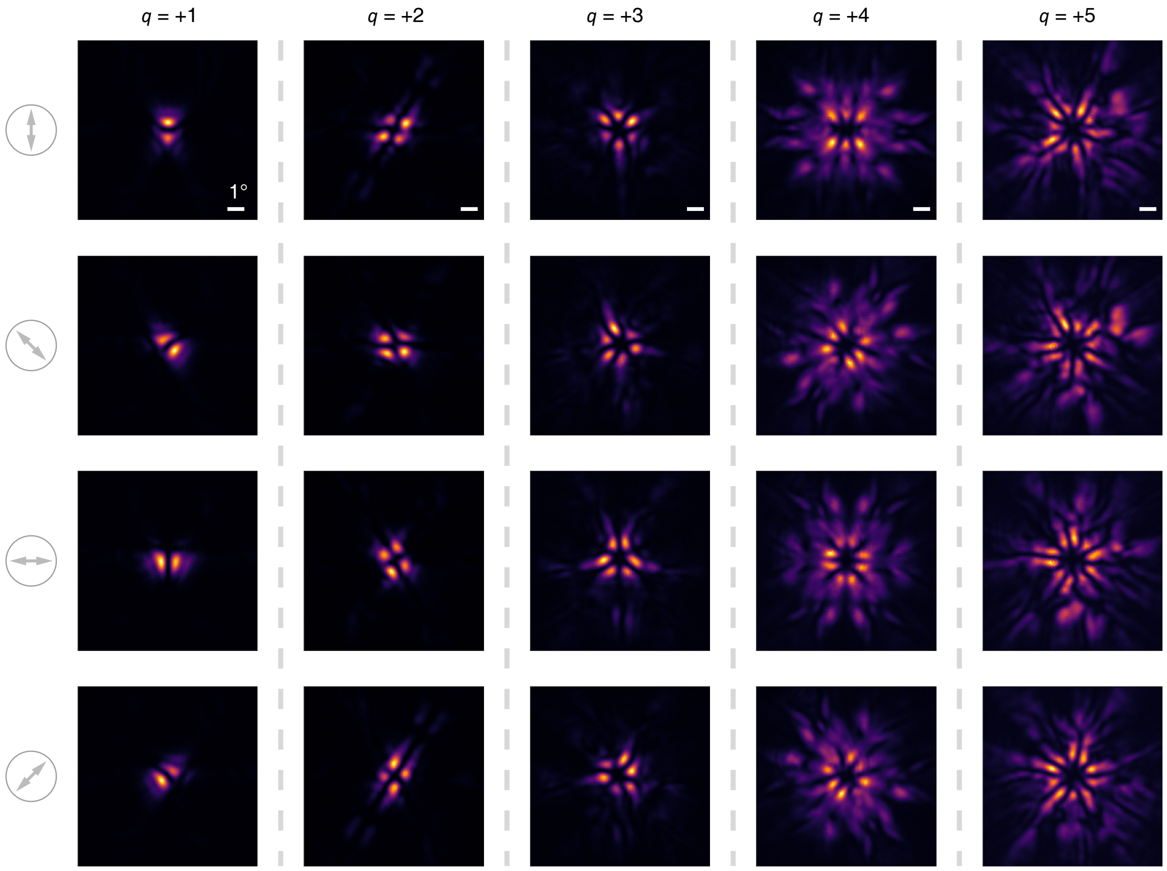

Figure S12: Measured polarization-resolved lasing images in momentum space with topological charges ranging from +1 to +5. The left grey arrows show directions of the analyzed polarizations.

Section 9. More details on the vectorial lasing characteristics

1. Measured Stokes parameter distribution

To quantify the clarity of the polarization state, we further analyzed the spatial distributions of the Stokes parameters based on the measured lasing profiles under linear polarizations (Section 8) of 0° (I_H), ±45° (I_D/I_A), and 90° (I_V). The Stokes parameters were calculated using the following relations:

$$S_0 = I_H + I_V, \quad S_1 = I_H - I_V, \quad S_2 = I_D - I_A, \quad |S_3| = (S_0^2 - S_1^2 - S_2^2)^{\frac{1}{2}}.$$

Figure S13 presents the normalized distributions of S_0, S_1/S_0, S_2/S_0, and $|S_3|/S_0$ for the vectorial lasing modes carrying different topological charges q. As $|q|$ increases, the spatial distributions of the S_1/S_0 and S_2/S_0 continue to exhibit clear angular alternations and consistent with the designed topological charges, indicating that the generated vectorial lasing modes retain distinct and accurate topological-charge information even at higher orders. In addition, the modes with negative charges generally display clearer and more uniform angular alternation.

Meanwhile, the distributions of $|S_3|/S_0$ suggest an overall tendency toward stronger circular polarization with increasing $|q|$, and this trend is more pronounced for positive charges. But it should be noted that some seemingly high values of $|S_3|/S_0$ occur in low-intensity regions, where the signal becomes comparable to the background fluorescence and both S_1/S_0 and S_2/S_0 are simultaneously small. In such cases, the normalization in the definition of $|S_3|/S_0$ may artificially amplify its value, leading to an overestimation of the local circular polarization.

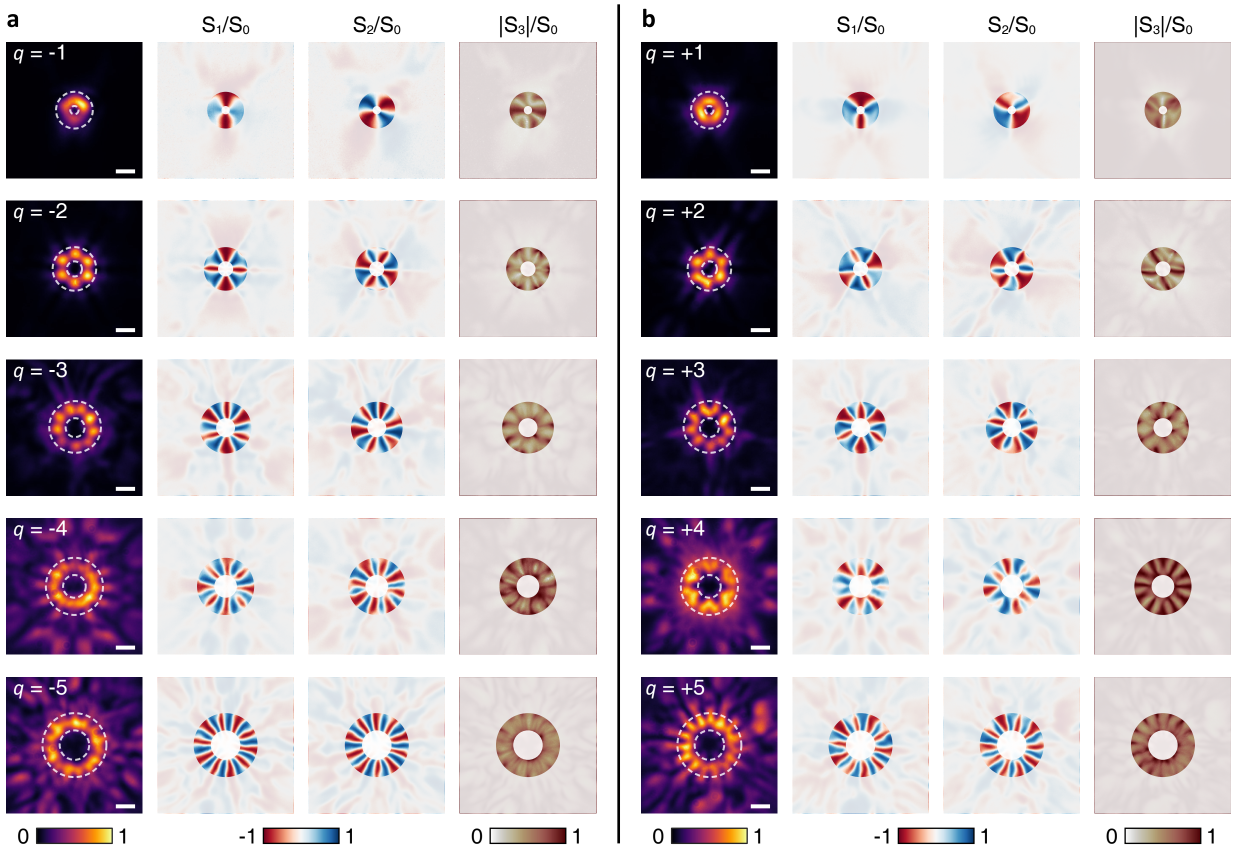

Figure S13: **a-b**, Measured lasing images and Stokes parameters with topological charges ranging from −1 to −5 (**a**) and +1 to +5 (**b**). The dashed circles are plotted to highlight the vectorial lasing profiles we focus on. Scale bar, 1°.

2. Measured divergence angle

From the measured lasing images shown in Figs.4-5 of the main text, it can be observed that the divergence angle of the emitted vectorial lasing tends to increase with the order of the topological charge. The divergence angles were extracted from the measured intensity profiles of each vectorial lasing mode, as summarized in Figure S14.

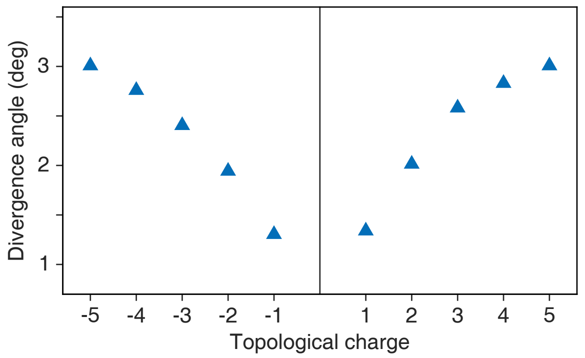

Figure S14: Measured divergence angle of the vectorial lasing with various topological charges.

3. Light-light curve

To discuss the lasing efficiency, we fabricated a new series of PhC structures with various combinations on the same substrate and characterized their lasing performance. The light–light curves for vectorial lasing modes with negative and positive charges are shown in Figure S15. The lasing efficiencies and thresholds are generally comparable, and they do not exhibit a clear or systematic trend as the topological charge varies.

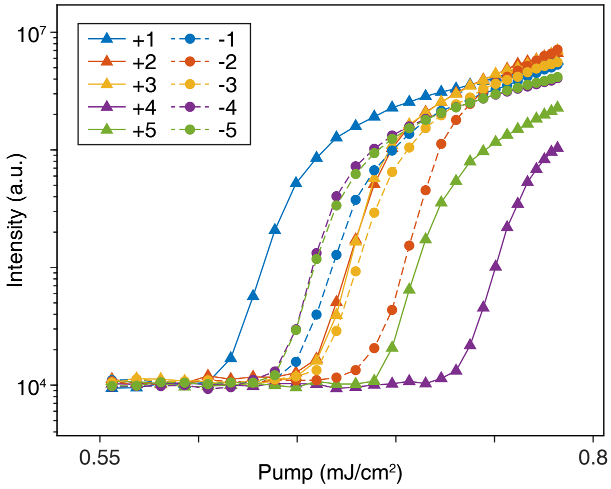

Figure S15: Light–light curves of vectorial lasing modes as functions of pump intensity.

4. Lasing wavelength

We also measured the lasing wavelengths of the newly fabricated compound cavities supporting vectorial lasing with different topological charges, along with a single quasi-BIC PhC slab ($\theta = 0°$) on the same substrate, enabling a direct comparison of their lasing wavelengths. As shown in Figure S16**a**, the single quasi-BIC slab lases at 863.03 nm, while the angle-resolved spectra of the vectorial modes (Figure S16**c**) lie slightly on the longer-wavelength side but remain very close. These results confirm that the vectorial lasing modes remain spectrally tied to the band-edge quasi-BIC.

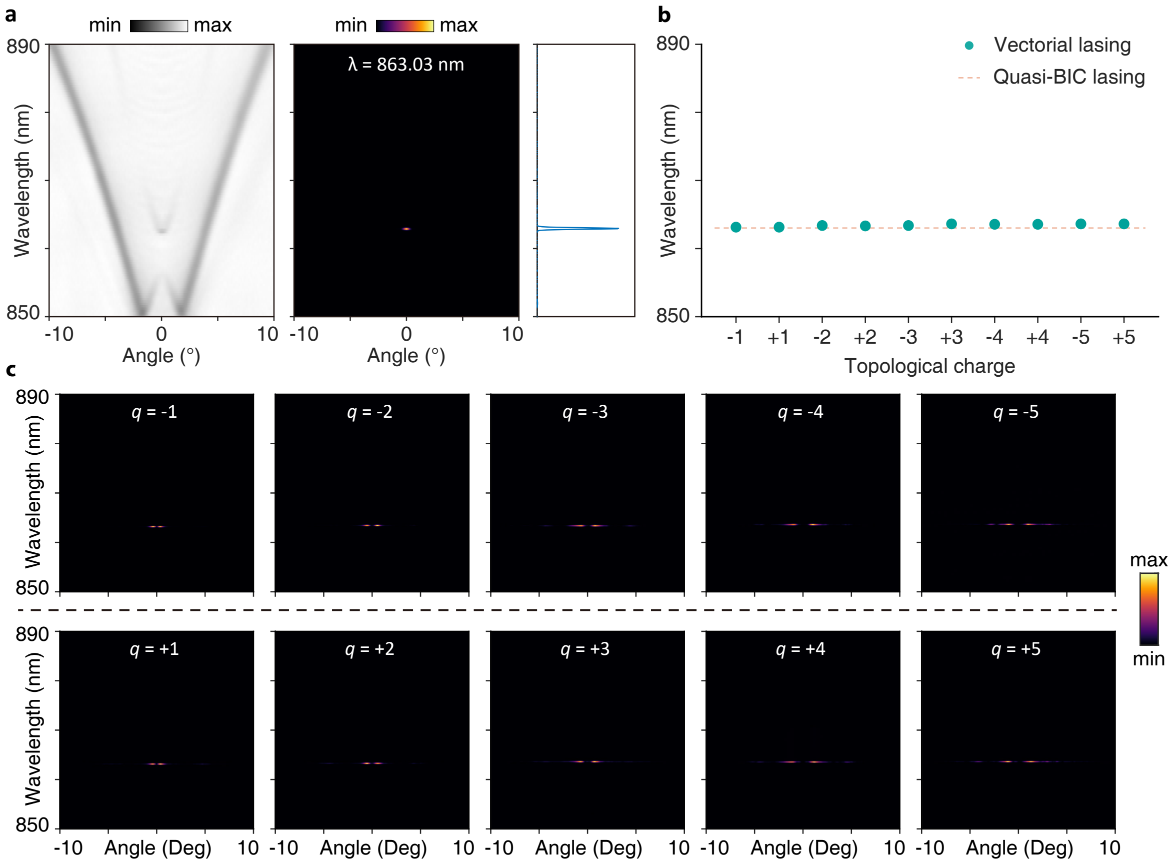

Figure S16: **a**, Measured transmittance spectra (s-polarized) and lasing spectra of the single quasi-BIC PhC slab with $\theta = 0°$. **b**, Comparison of the measured lasing wavelength between the quasi-BIC lasing and vectorial lasing with different topological charges. **c**, Measured angle-resolved lasing spectra (above threshold) of the compound cavities supporting vectorial lasing with different topological charges at the same substrate.

Section 10. Discussions on the compound cavity mode for vectorial lasing

To further verify that the vectorial lasing is originated from the cavity mode in structure center, we fabricated two compound structures with identical slab arrangements but different central configurations: (**a**) without a central blank, and (**b**) with an 8-μm-diameter central blank intentionally introduced to disrupt the compound cavity, as shown in Figure S17. These two structures are fabricated together on the same substrate to enable direct comparison of their emission behavior and to investigate the origin of the vectorial lasing modes. Under identical optical pumping conditions (pump laser spot diameter ~40 μm), the structure with the 8 μm blank exhibited drastically different emission behavior. The measured angle-resolved photoluminescence (PL) spectra and polarization-resolved lasing images confirmed the presence of vectorial lasing modes with a topological charge of −4 in the structure shown in Figure S17**a**. In contrast, for the structure with the 8 μm central blank (Figure S17**b**), only incoherent fluorescence with a broad spectral distribution was observed, as the central blank disrupted the cavity required for vectorial lasing.

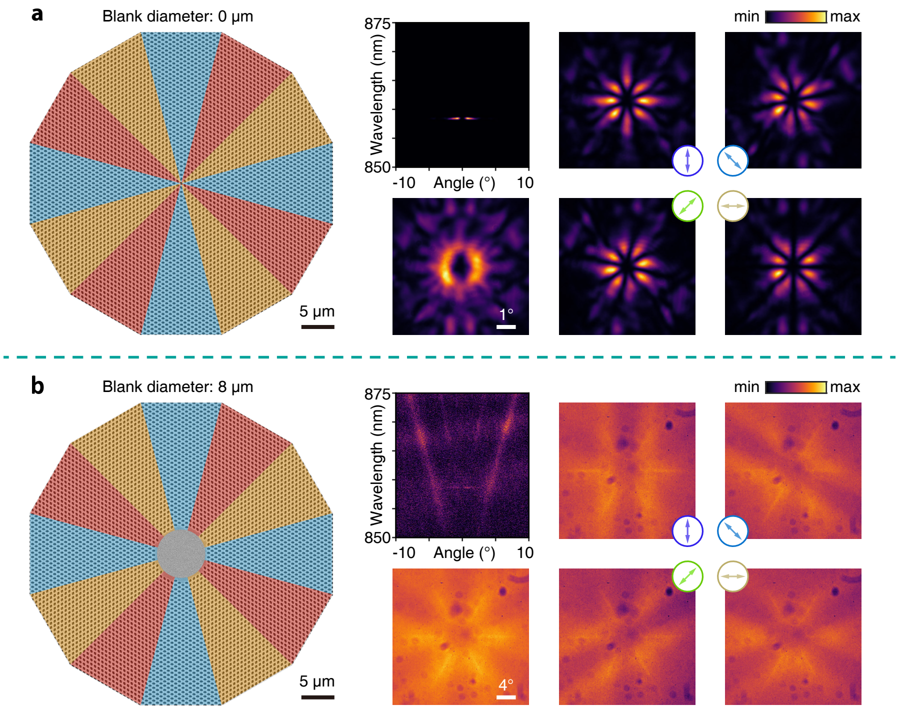

Figure S17: Experimental demonstration of the compound cavity mode. **a-b**, The compound structures are designed to generating vectorial lasing with −4 charge, while we induced the circular central blank with diameter of 8 μm to disrupt the central cavity in **b**. The right panels show their angle-resolved PL spectra and lasing images.

In contrast to the hollow-centered compound cavity in Figure S17**b**, which severely disrupts the central cavity mode, the compound structure formed by combining quasi-BIC PhC slabs exhibits a certain degree of robustness against structural perturbations. For example, we modified the compound cavity by replacing the hollow center with unperturbed, homogeneous circular PhC cavities (triangular lattice of circular holes, possessing C_6 symmetry) of different diameters. The top panels of Figures S18**a**–**d** show SEM images of the fabricated structures near the cavity center on the same substrate. Structure of (**a**) corresponds to the original compound cavity supporting vectorial lasing with −4 charge, whereas in structures of (**b-d**), the central region is replaced with C_6-symmetric PhC slabs with diameters of 8, 16, and 24 μm, respectively (highlighted in purple). All structures were excited with the same optical pump spot (diameter ~40 μm). For structures of (**a**), (**b**), and (**c**), stable vectorial lasing is observed, and polarization-resolved measurements confirm that their topological charges all remain −4. However, for structure of (**d**), lasing is no longer observed under the same pumping conditions, nor does it appear even when the pump power is further increased.

These observations show that a small C_6-symmetric insert will not significantly disturb the cavity mode. In our low-index Si_3N_4 photonic platform, with an overall cavity diameter of 150 μm and a pump spot

diameter of ~40 μm, central inserts up to 16 μm in diameter do not noticeably alter the vectorial lasing behavior. In Figure S18**d**, the 24-μm C_6-symmetric insert disrupts the vectorial cavity mode supported by the surrounding compound structure, yet remains too small to sustain its own BIC lasing, and consequently no lasing is observed. As shown in Figure S19, isolated C_6-symmetric PhC cavities remain unable to support lasing even at diameters as large as 32 μm.

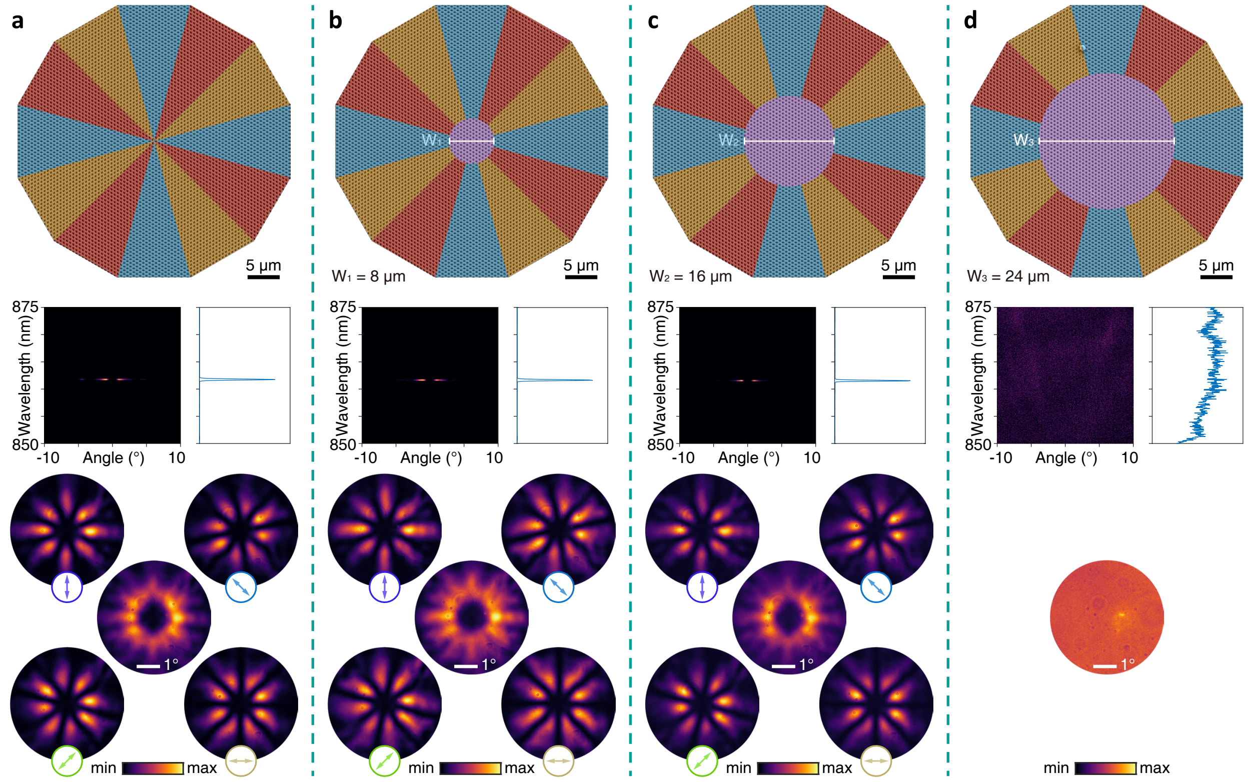

Figure S18: Measured lasing images of the compound PhC structures with different central regions. The top panels present the SEM images of fabricated compound structures, where the purple regions correspond to the unperturbed homogeneous round PhC cavities. Their lasing spectra and images are presented in the middle and bottom panels, respectively.

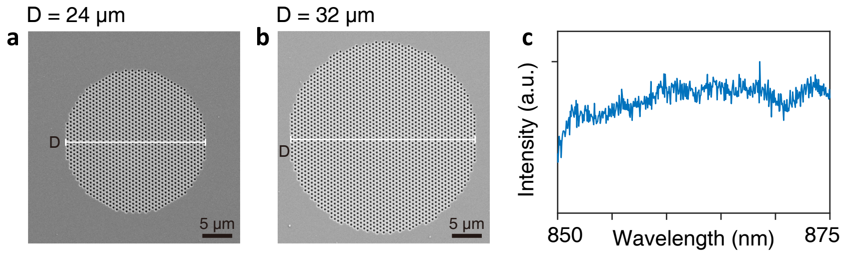

Figure S19: **a-b**, SEM images of the isolated C_6-symmetric PhC cavities. **c**, Emission characteristics of the isolated C_6-symmetric PhC cavity with diameter of 32 μm.

Section 11. Discussions on the topological polarization configurations of the lasing modes

Our work utilizes the Möbius-like correspondence of q-BICs to construct cavity mode with designable topological charges. In this framework, one complete loop of the Möbius-like correspondence contributes to a topological charge of $|q| = 1$, and the lasing topological charge is controlled by the orientation and repeating number of the loop. When the compound-cavity configuration is modified such that the original angular repetition based on the Möbius-like correspondence is disrupted, the resulting lasing behaviors is no longer intuitively predictable. Here, we carried out additional experiments as examples and provided more discussions below:

Figure S20**a** presents the same $q = +4$ cavity configuration as that in Fig. 4 of the main text, where the resulting vectorial lasing exhibits a polarization configuration consistent with a +4 polarization vortex.

In Figure S20**b**, one of the blue regions in the structure of Figure S20**a** is removed. In this case, the remaining structure can no longer support lasing. This indicates that the removal severely disrupts the integrity of the compound cavity, preventing the formation of a cavity mode required for lasing.

Figures S20**c**–**d** replace the blue sector on the right-hand side of the structure in Figure S20**a** with other different PhC sector. Although the resulting lasing images retain certain polarization features, the polarization-vortex configurations are strongly perturbed and highly asymmetric, and therefore cannot be unambiguously assigned a well-defined polarization topological charge.

All these structures were fabricated on the same substrate and optically pumped under identical conditions (pump laser spot diameter ∼40 μm) to enable a direct comparison.

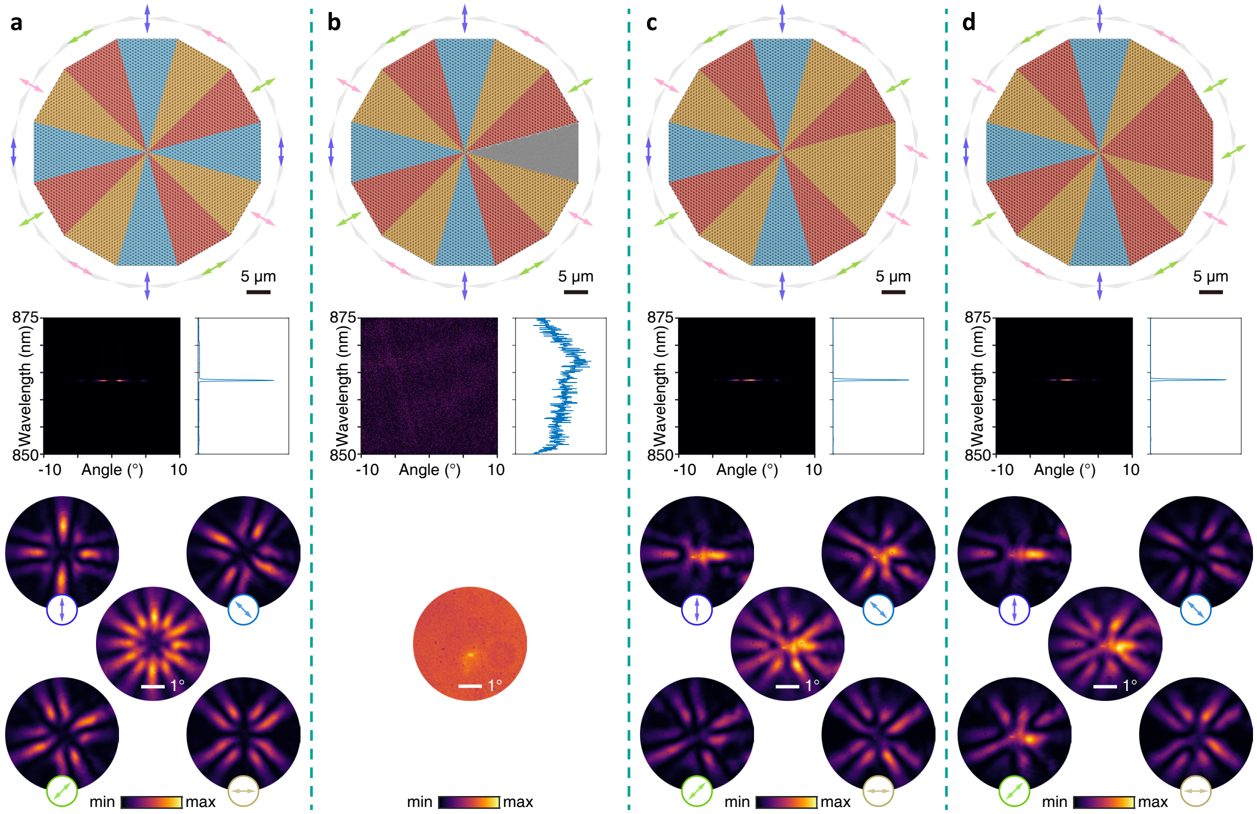

Figure S20: Comparison of emission characteristics between different compound structures. The top panels present the SEM images of the fabricated compound structures, with different colors highlighting the orientation of the elliptical holes. And the colored arrows outside indicate polarization states of the composed q-BIC PhC slabs. Their lasing spectra are presented in the middle panels. The bottom panels present the corresponding lasing images, where the total intensity distributions are plotted in the top left corner.

These results indicate that the cavity mode cannot be simply interpreted as the direct interference of different PhC sectors. To further demonstrate this point, we constructed another compound cavity that does not follow the repeating arrangement of Möbius-like correspondence (Figure S21). In this case, the resulting

polarization distribution no longer matches that expected from direct interference among the sectors. For example, the PhC sectors highlighted in blue support quasi-BIC modes with vertically oriented polarization. When the lasing image is analyzed using a polarizer aligned to this polarization, no pronounced lasing intensity is observed at the corresponding azimuthal angles.

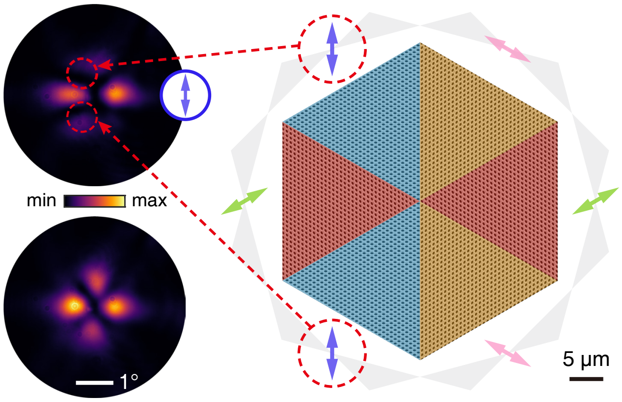

Figure S21: Emission characteristics of the compound cavities with modified combinations.

Section 12. Constraints of structural symmetry on the topological charges of the symmetry-protected BICs at Γ point

The non-trivial lasing profiles are dominated by momentum-space topological vortex configurations of BICs, which are protected by real-space structural symmetries. More specifically, the topological charge of BICs is both protected and constrained by the structure's rotational symmetries [5, 6]. Thus, lasing achieved via these symmetry-protected BICs in previous studies has been limited to a few specific topological charges, primarily -1 and $+1$, with rare instances of -2. To give more detailed description, we give a brief summary about the topological charge of BICs based on the symmetry analysis based on the group theory [6], as shown in Figure S22.

Symmetry	Representations	Charges	Allowed charges	Realized charges
C_2	A	$2n + 1$	±1, ±3, ±5, ...	-1 Phys. Rev. Lett. 133, 213802 (2024), etc.<br>+1 Nature Photonics 12, 397-401 (2018), etc.
C_3	A	$3n + 1$	+1, -2, +4, ...	+1 Phys. Rev. Lett. 125, 053902 (2020), etc.
C_4	A	$4n + 1$	+1, -3, +5, ...	+1 Science 367, 1018-1021 (2020), etc.
	B	$4n - 1$	-1, +3, -5, ...	-1 Advanced Materials 35, 2207430 (2023), etc.
C_6	A	$6n + 1$	+1, -5, +7, ...	+1 Physical Review B 109, 075134 (2024), etc.
	B	$6n - 2$	-2, +4, -8, ...	-2 Phys. Rev. Lett. 125, 053902 (2020), etc.
	E_2	$6n - 2$	-2, +4, -8, ...	-2 Phys. Rev. Lett. 133, 036201 (2024), etc.

Figure S22: All possible and realized topological charges of the symmetry-protected BICs at Γ point. Here $n = 0, \pm1, \pm2...$ is an integer.

For a PhC slab with certain rotational symmetry, each mode at the Γ point corresponds to an irreducible representation of the symmetry group. Around the Γ point, the polarization state follows $\mathcal{R}\mathbf{u} = c\mathbf{u}$, where $\mathbf{u}$ represents the polarization state, $\mathcal{R}$ is the rotational operator and c is the character of the corresponding representation. This relation ensures the robust existence of symmetry-protected BICs, but also constrains their topological polarization configurations. Here, we also give a specific example of a BIC with -2 topological charge to illustrate the symmetry constraint, as shown in Figure S23.

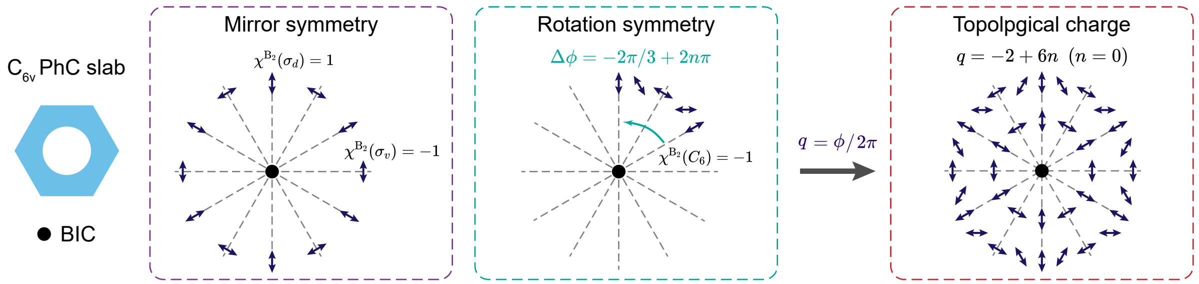

Figure S23: Symmetry analysis of the topological polarization configurations of the -2-charged BIC in C_{6v}-symmetric PhC slab.

For the C_{6v}-symmetric PhC slab of triangular lattice, the BIC of -2 charge corresponds to the B_2 representation of the C_{6v} point group. As illustrated in the purple box of Figure S23, the presence of mirror symmetry requires that the polarization states in the mirror plane (marked as dashed lines) must be linearly polarized, and their polarization orientations are determined by the characters of mirror operations (σ_v and σ_d). For the σ_v operation, the character $\chi^{B_2}(\sigma_v) = -1$, indicating that the polarization states in these mirror planes change signs under the mirror operation, hence their polarization orientations need to by perpendicular to the mirror planes. While for the σ_d operation, the character $\chi^{B_2}(\sigma_d) = 1$. Thus, the

polarization states in the σ_d mirror planes are parallel to the planes. For the C_6 operation, the character $\chi^{B_2}(C_6) = -1$. This means that when a polarization state is rotated 60° around the z-axis, it will have the opposite sign compared to the original polarization state. Consequently, the polarization orientation angle ϕ should have a variation of $2n - 2\pi/3$ (where n is an integer) for a rotation of 60°, as illustrated in the cyan box of Figure S23. For a complete rotation around the z-axis, the total angle change ϕ becomes $12n\pi - 4\pi$, resulting in a topological charge of $q = 6n - 2$, as shown in the red box of Figure S23 for $n = 0$.

It should be noted that via these symmetry analyses we can only obatain an n-related constraint for the allowed topological charges of BICs. The PhC slabs with a definite symmetry can still support BICs with different topological charges. Hence, in most commonly used method at present, the exact topological charge of a BIC is calculated by numerical simulations with the practical considerations of structural parameters and material parameters. This also contributes to the limitations of controllability and designability in tradiational BIC lasing. Moreover, even though symmetry analysis has offered the allowed topological charges, the currently observed topological charges of BICs are still confined to a narrow range, as presented in right part of Figure S22.

Section 13. Comparison of the Dirac-vortex cavity and the compound cavity in this work

The approach constructing quasi-BICs to form compound cavity for vectorial lasing differs from the Dirac-vortex cavity method in the following key perspectives:

a. From the physical principles of cavity design to realize vectorial lasing:

The Dirac-vortex cavities used to generate vectorial lasing are based on two-dimensional (2D) mid-gap defect cavities [7]. To realize such a topological mid-gap mode, one must first open a vortex bandgap of a double Dirac cone dispersion by specific symmetry breaking. As illustrated in the left panels of Figure S24 ("Band structures" and "Structure design"), utilizing the generalized Kekulé modulation for symmetry breaking, a Dirac-mass vortex around a central defect is constructed, giving rise to a photonic realization of the Jackiw-Rossi mid-gap mode for vectorial lasing. In contrast, our approach begins with a non-degenerate BIC at the Γ point. Through controlled symmetry breaking, we transform this BIC into a quasi-BIC with linear eigen-polarization, as illustrated in the right panels of Figure S24 ("Band structures" and "Structure design"). By in-plane arranging multiple PhC slabs, each supporting quasi-BICs with different eigen-polarizations, we construct the compound cavities to generate vectorial lasing with designable topological charges.

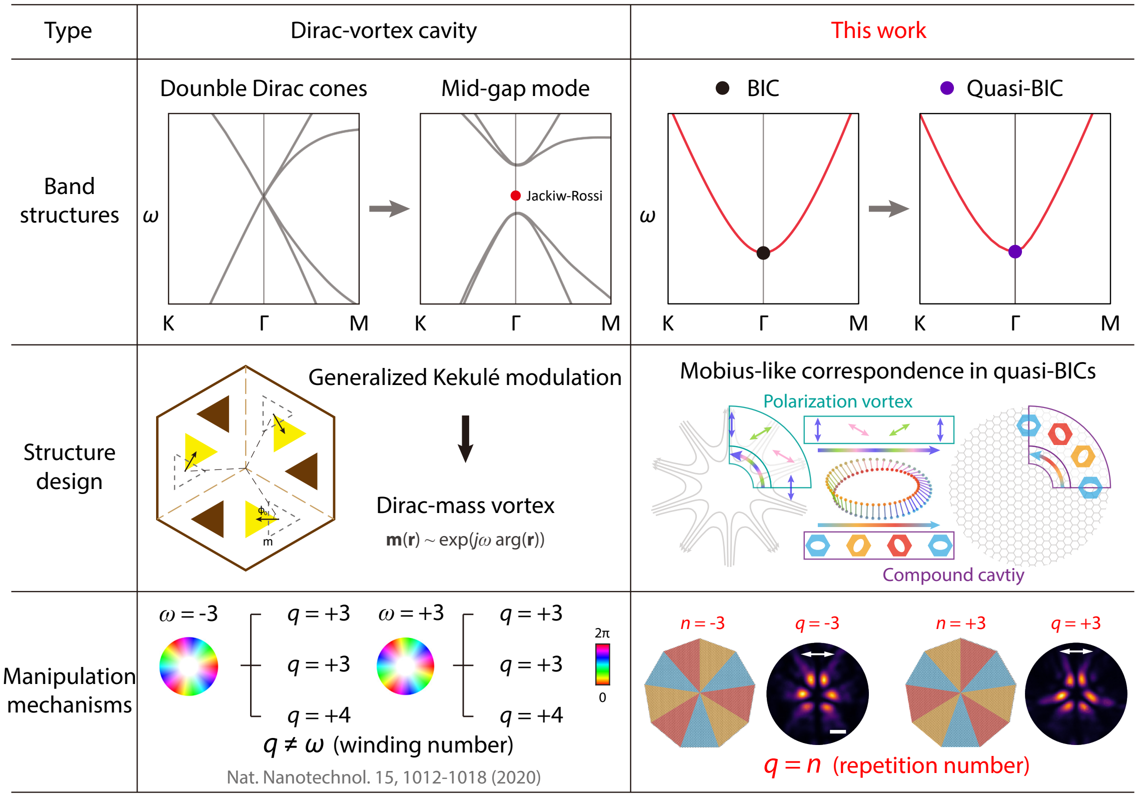

Figure S24: Comparison of the Dirac-vortex cavity and the compound cavity in this work.

b. From the manipulating mechanisms of topological charge q:

The Dirac-vortex cavities and our methodology also show differences in the manipulating mechanisms for topological charge q, which are especially presented in the correspondence between real-space winding number ω (Dirac-vortex cavity) or repetition number n (compound cavity in this work) and topological charge q of realized vectorial lasing. The mid-gap modes of Dirac-vortex cavities are vortex solutions to the Dirac equations with mass vortices, where the Dirac-mass winding number serves as the key parameter governing the topological charges of the lasing profiles. For the reported work of Dirac-vortex cavities, as presented in the left panel of Figure S24 ("Manipulation mechanisms"), a given winding number ω does not uniquely determine q. For example, a Dirac-vortex cavity with $\omega = +3$ can generate lasing profiles with $q = +3$ or $q = +4$, and a cavity with $\omega = -3$ may also support lasing with $q = +3$ or $q = +4$. Thus, the topological charge q of lasing modes in Dirac-vortex cavities is not strictly equal to the winding number ω. In contrast, our approach exploits the Möbius-like correspondence in quasi-BICs to directly construct

the desired topological charge. The angular arrangement of quasi-BIC PhC slabs uniquely determines the topological charge of the cavity mode, thereby establishing a one-to-one correspondence between compound cavities and their lasing topological charge. Consequently, the repetition number n of our compound cavities exactly equals the lasing topological charge q, as illustrated in the right panel of Figure S24 ("Manipulation mechanisms").

To summarize, our methodology differs from Dirac-vortex cavities in these critical aspects: physical principles (quasi-BICs vs. topological mid-gap modes), manipulation mechanisms (Möbius-like correspondence, $q = n$ vs. Dirac-mass winding number, $q \neq \omega$). In contrast to previously reported Dirac-vortex studies, our work offers a new approach to achieve vectorial lasing and enabling the on-demand construction of lasing topological charges.

Section 14. Comparison of the photonic disclination cavity and the compound cavity in this work

The approach constructing quasi-BICs to form compound cavity for vectorial lasing differs from the photonic disclination cavity method in the following key perspectives:

a. Designable control over the topological charge

First, we would like to give more detailed discussion on the designable control over the topological charge of the emitted vectorial lasing modes. In disclination-based nanolasers, the topological charge of the emitted vector beam is fundamentally determined by the angular momentum index l as [8]:

$$|q| = |1 - |l||.$$

As presented in the first line of Figure S25, Hwang *et al.* demonstrated the generation of $q = 1$ using the $l = 0$ mode and $q = -1$ using the $|l| = 2$ mode) [8], and Shin *et al.* achieved a higher-order topological charge of $|q| = 2$ by utilizing the $l = 3$ mode [9].

In our work, by leveraging the Möbius-like correspondence, we establish a one-to-one mapping ($q = n$) between the integer topological charge q and the number of angularly repeated sectors n. This mechanism enables the deterministic design of topological charges simply by reconfiguring the sectoral arrangement, and we have experimentally realized vectorial lasing over a wider range, from -5 to $+5$.

	Photonic disclination cavity<br>Nature Photonics 18, 286-293 (2024)<br>ACS Photonics 12, 2530-2537 (2025)	This work
Designable control over topological charge	$\|q\| = \|1 - \|l\|\|$	$q = n$<br>(n: repetition number)
Mechanism for achieving high-Q modes	Defect mode<br>Bandgap	Q-BIC<br>Band edge
Beam divergence	$\|q\| = 1$<br>Divergence angle: 34.5°<br>Nature Photonics 18, 286-293 (2024)	$\|q\| = 1$<br>Divergence angle: < 2°

Figure S25: Comparison of vectorial lasing realized through photonic disclination cavity and our work.

b. Mechanism for achieving high-Q modes

Photonic disclination nanolasers rely on topological defect resonant modes that reside inside the photonic bandgap (PBG) of the bulk lattice. These localized states are prevented from coupling to the surrounding continuum, which enables high quality (Q) factors to be preserved even in ultra-small cavities. By contrast, the lasing modes in our work originate from band-edge quasi-BIC states with high-Q factors. Rather than opening a photonic bandgap, our approach exploits symmetry engineering of the PhC structure to generate quasi-BIC modes with high Q factors and tunable polarization states.

c. Beam divergence

As the two approaches rely on fundamentally different confinement mechanisms, their far-field characteristics also differ significantly. For example, we compare the two approaches for the vectorial lasing with same topological charge $|q| = 1$. The lasing modes in ultra-small disclination cavities exhibit a large divergence angle (measured to be 34.5° [8]). In our work, the resulting emission displays a much smaller divergence angle ($< 2°$). This highly directional output is a benefit of the quasi-BIC mechanism.

To summarize, beyond the designable control of topological charge, our work also differs from photonic disclination nanolasers in the mechanism for achieving high-Q modes (band-edge quasi-BIC modes vs. photonic-bandgap defect modes), which in turn leads to vectorial lasing with a significantly smaller divergence angle.

References

[1] Akahane, Y., Asano, T., Song, B.-S. & Noda, S. High-q photonic nanocavity in a two-dimensional photonic crystal. *Nature* **425**, 944–947 (2003).

[2] Yang, Z.-Q., Shao, Z.-K., Chen, H.-Z., Mao, X.-R. & Ma, R.-M. Spin-momentum-locked edge mode for topological vortex lasing. *Physical Review Letters* **125**, 013903 (2020).

[3] Hwang, M.-S. *et al.* Ultralow-threshold laser using super-bound states in the continuum. *Nature Communications* **12**, 4135 (2021).

[4] Zhang, Y. *et al.* Momentum-space imaging spectroscopy for the study of nanophotonic materials. *Science Bulletin* **66**, 824–838 (2021).

[5] Hsu, C. W., Zhen, B., Stone, A. D., Joannopoulos, J. D. & Soljačić, M. Bound states in the continuum. *Nature Reviews Materials* **1**, 1–13 (2016).

[6] Wang, J. *et al.* Optical bound states in the continuum in periodic structures: mechanisms, effects, and applications. *Photonics Insights* **3**, R01–R01 (2024).

[7] Gao, X. *et al.* Dirac-vortex topological cavities. *Nature Nanotechnology* **15**, 1012–1018 (2020).

[8] Hwang, M.-S. *et al.* Vortex nanolaser based on a photonic disclination cavity. *Nature Photonics* **18**, 286–293 (2024).

[9] Shin, C., Kim, H.-R., Kim, D. & Park, H.-G. Photonic disclination nanolaser with a high topological charge of 2. *ACS Photonics* **12**, 2530–2537 (2025).
